# Late recovery from chronic arginine vasopressin deficiency: a multicenter retrospective case-control study

**DOI:** 10.1210/jendso/bvag211

**Published:** 2026-09-16

**Authors:** Clara O Sailer, Francesca Galbiati, Anna Aulinas, Maged Muhammed, Regine Boutin, Jenna Bourassa, Cihan Atila, Julia Crowley, Allie Dayno, Hannah Pearlstein, Lisa B Nachtigall, Karen K Miller, Nicholas A Tritos, Ursula B Kaiser, Shana E McCormack, Mirjam Christ-Crain, Laura E Dichtel, Elizabeth A Lawson

**Affiliations:** Section of Neuroendocrinology and Reproduction, Division of Endocrinology, Diabetes, and Metabolism, Department of Medicine, Mass General Brigham, Harvard Medical School, Boston, MA 02114, USA; Mass General Brigham Pituitary Center, Mass General Brigham, Boston, MA 02114, USA; Section of Neuroendocrinology and Reproduction, Division of Endocrinology, Diabetes, and Metabolism, Department of Medicine, Mass General Brigham, Harvard Medical School, Boston, MA 02114, USA; Mass General Brigham Pituitary Center, Mass General Brigham, Boston, MA 02114, USA; Department of Endocrinology and Nutrition, Hospital de la Santa Creu i Sant Pau, EndoERN, IR-Sant Pau, CIBERER Unidad 747 (ISCIII), 08041 Barcelona, Spain; Faculty of Medicine, University of Vic—Central University of Catalonia, 08500 Vic, Spain; Section of Neuroendocrinology and Reproduction, Division of Endocrinology, Diabetes, and Metabolism, Department of Medicine, Mass General Brigham, Harvard Medical School, Boston, MA 02114, USA; Mass General Brigham Pituitary Center, Mass General Brigham, Boston, MA 02114, USA; Section of Neuroendocrinology and Reproduction, Division of Endocrinology, Diabetes, and Metabolism, Department of Medicine, Mass General Brigham, Harvard Medical School, Boston, MA 02114, USA; Mass General Brigham Pituitary Center, Mass General Brigham, Boston, MA 02114, USA; Section of Neuroendocrinology and Reproduction, Division of Endocrinology, Diabetes, and Metabolism, Department of Medicine, Mass General Brigham, Harvard Medical School, Boston, MA 02114, USA; Department of Endocrinology, Diabetology and Metabolism, University Hospital Basel, University of Basel, 4031 Basel, Switzerland; Neuroendocrine Center, Division of Endocrinology and Diabetes, Children's Hospital of Philadelphia, Philadelphia, PA 19104, USA; Neuroendocrine Center, Division of Endocrinology and Diabetes, Children's Hospital of Philadelphia, Philadelphia, PA 19104, USA; Neuroendocrine Center, Division of Endocrinology and Diabetes, Children's Hospital of Philadelphia, Philadelphia, PA 19104, USA; Section of Neuroendocrinology and Reproduction, Division of Endocrinology, Diabetes, and Metabolism, Department of Medicine, Mass General Brigham, Harvard Medical School, Boston, MA 02114, USA; Mass General Brigham Pituitary Center, Mass General Brigham, Boston, MA 02114, USA; Section of Neuroendocrinology and Reproduction, Division of Endocrinology, Diabetes, and Metabolism, Department of Medicine, Mass General Brigham, Harvard Medical School, Boston, MA 02114, USA; Mass General Brigham Pituitary Center, Mass General Brigham, Boston, MA 02114, USA; Section of Neuroendocrinology and Reproduction, Division of Endocrinology, Diabetes, and Metabolism, Department of Medicine, Mass General Brigham, Harvard Medical School, Boston, MA 02114, USA; Mass General Brigham Pituitary Center, Mass General Brigham, Boston, MA 02114, USA; Section of Neuroendocrinology and Reproduction, Division of Endocrinology, Diabetes, and Metabolism, Department of Medicine, Mass General Brigham, Harvard Medical School, Boston, MA 02114, USA; Mass General Brigham Pituitary Center, Mass General Brigham, Boston, MA 02114, USA; Neuroendocrine Center, Division of Endocrinology and Diabetes, Children's Hospital of Philadelphia, Philadelphia, PA 19104, USA; Department of Endocrinology, Diabetology and Metabolism, University Hospital Basel, University of Basel, 4031 Basel, Switzerland; Section of Neuroendocrinology and Reproduction, Division of Endocrinology, Diabetes, and Metabolism, Department of Medicine, Mass General Brigham, Harvard Medical School, Boston, MA 02114, USA; Mass General Brigham Pituitary Center, Mass General Brigham, Boston, MA 02114, USA; Section of Neuroendocrinology and Reproduction, Division of Endocrinology, Diabetes, and Metabolism, Department of Medicine, Mass General Brigham, Harvard Medical School, Boston, MA 02114, USA; Mass General Brigham Pituitary Center, Mass General Brigham, Boston, MA 02114, USA

**Keywords:** arginine vasopressin deficiency, central diabetes insipidus, pituitary hormone deficiency, desmopressin

## Abstract

**Context:**

Arginine vasopressin deficiency (AVP-D), caused by hypothalamic/pituitary damage from tumor or surgery, can be transient or chronic. Chronic AVP-D is thought to be permanent though emergent research indicates the possibility of late recovery. Little is known about clinical characteristics that predict late recovery from chronic AVP-D.

**Objective:**

Describe characteristics associated with late recovery from chronic AVP-D.

**Design:**

Multicenter, retrospective case-control study of AVP-D Recovered (n = 18) vs AVP-D Non-recovered (n = 36; matched for sex and disease duration).

**Main outcome measures:**

Clinical characteristics assessed at hypothalamic/pituitary disease diagnosis, AVP-D diagnosis, AVP-D recovery, and most recent clinical evaluation. We also present characteristics of three pediatric cases with AVP-D recovery.

**Results:**

At diagnosis of hypothalamic/pituitary disease, AVP-D Recovered, compared to AVP-D Non-recovered, were older (45 ± 19 vs 33 ± 13 years, *P* = .006) and more likely to have functioning pituitary tumors (33.3% vs 8.3%, *P* = .05). Following diagnosis of AVP-D, AVP-D Recovered received lower total daily doses of desmopressin (100 [IQR; 50, 100] vs 200 [100, 200] µg oral or equivalent, *P* = .01). Recovery occurred after a median of 6.3 years [1.7, 12.0] and occurred earlier in those without hypothyroidism (*P* = .049) or adrenal insufficiency at diagnosis of AVP-D (*P* = .008).

**Conclusion:**

Our study supports the notion that chronic AVP-D may not be permanent across the lifespan. Continued requirement for desmopressin, particularly in those with a history of functioning pituitary tumors, low doses of desmopressin, and without concurrent hypothyroidism or adrenal insufficiency at diagnosis, should be regularly reassessed.

Arginine vasopressin deficiency (AVP-D), formerly known as central diabetes insipidus (1), is a rare condition characterized by insufficient secretion of arginine vasopressin (AVP) from the hypothalamic-neurohypophyseal tract (2, 3). Reduced circulating AVP concentration leads to hypotonic polyuria and compensatory polydipsia, which is treated with AVP replacement using desmopressin (DDAVP, 1-deamino-8-D-arginine vasopressin) (3, 4). Acquired AVP-D occurs due to structural injury of the hypothalamus or neurohypophysis, which can result from primary (eg, craniopharyngioma, lymphoma) or secondary brain tumors (metastatic disease), neurosurgery, traumatic brain injury, inflammatory conditions, infiltrative disease, or apoplexy. The condition may be transient, lasting from days to weeks, or chronic (also described as persistent or permanent AVP-D), if persistent beyond 8 weeks to 6 months (5-7). One series noted AVP-D acutely after transsphenoidal surgery (TSS) in nearly 11% of 2529 patients, which persisted in 5% of cases, with a higher risk of chronic AVP-D after TSS for craniopharyngioma (27%) vs pituitary adenoma (3%) (8). Chronic AVP-D typically requires lifelong desmopressin replacement. However, cases of recovery from chronic AVP-D have been documented, suggesting that the term “permanent” AVP-D may be misleading.

Several case reports and small case series, most commonly in individuals with craniopharyngiomas, have described spontaneous recovery in individuals with chronic AVP-D (6, 9-11). In a study of 22 adults who underwent pituitary surgery, 20 had stalk dissection and all developed postoperative chronic AVP-D, with four (in whom stalk resection was more distal) subsequently recovering from AVP-D after a median time of 2.7 years (6). Similarly, in another series of 44 adults diagnosed with craniopharyngioma who developed postoperative AVP-D, seven adults recovered from AVP-D after more than six months (10). The only reported case of spontaneous recovery from chronic AVP-D in a child described a 16-year-old girl who developed AVP-D after surgery for pituitary apoplexy, and this resolved 1 year after diagnosis (9). These limited observations suggest that chronic AVP-D does not invariably persist for life. Identifying characteristics of individuals who recover from chronic AVP-D may help minimize patient burden, prevent treatment-related complications (eg, hyponatremia) during recovery, and provide insights into mechanisms of recovery.

Thus, we aimed to improve the understanding of factors associated with recovery from chronic AVP-D in a retrospective, multisite international cohort. First, we analyzed differences in clinical, biochemical, and imaging characteristics in those with chronic AVP-D due to hypothalamic and/or pituitary damage who experienced spontaneous recovery (AVP-D Recovered) vs those with similar AVP-D disease duration but no recovery (AVP-D Non-recovered). We hypothesized that AVP-D Recovered would present with less severe hypothalamic and pituitary injury, as reflected by smaller lesion size, relatively preserved pituitary function, and a lower desmopressin dose compared with AVP-D Non-recovered. Second, for AVP-D Recovered, we described clinical, biochemical, and imaging characteristics at the time of recovery and evaluated predictors of earlier recovery from AVP-D. Third, since recovery from chronic AVP-D is rare and to our knowledge only one pediatric case has been published, we describe the clinical course of three children who recovered from chronic AVP-D.

## Materials and methods

### Study design and participants

Our retrospective international multicenter case-control study included participants from Massachusetts General Hospital and Brigham and Women's Hospital (Mass General Brigham (MGB); Boston, MA, United States), the Children's Hospital of Philadelphia (CHOP; Philadelphia, PA, United States), and the University Hospital of Basel (Basel, Switzerland). The Institutional Review Board (IRB) of MGB approved the study. Electronic medical record review was considered IRB exempt by the CHOP IRB and the Ethics Committee of Northwest and Central Switzerland. Informed consent was waived as only existing health record data were used, and participants who had previously declined the use of their health records for research were excluded. To identify cases of late recovery, investigators contacted practicing endocrinologists specializing in pituitary disorders at participating centers and requested referral of potential cases. Identified cases underwent detailed review by study investigators, including assessment of clinical notes, laboratory data, treatment history, and follow-up documentation to confirm recovery status. Individuals with infundibulo-neurohypophysitis were excluded because of the fluctuating nature of the disease. Recovery from AVP-D was defined as successful discontinuation of desmopressin after at least 6 months of treatment, without subsequent reinstitution of therapy during follow-up. Two controls (AVP-D Non-recovered) matched for sex and AVP-D disease duration (± 5 years) were identified for each case. Duration of AVP-D was defined as the time from the date of AVP-D diagnosis to the date of recovery in AVP-D Recovered and to the date of the most recent clinical evaluation for AVP-D Non-recovered. We adhered to the STROBE (Strengthening the Reporting of Observational Studies in Epidemiology) guidelines for reporting observational case-control studies. The three children with AVP-D recovery were not included in the case-control study given the small sample and lack of comparability with the adult cohort. Instead, these cases are reported descriptively.

### Methods

For each participant, medical history and eligibility were retrospectively reviewed using electronic health records, including discharge summaries, clinic and procedure notes, laboratory results, pathology, and radiology reports. Extracted information included (1) demographic data (age, sex, ethnicity, and race); and (2) clinical characteristics, including at (a) diagnosis of hypothalamic/pituitary disease, (b) diagnosis of AVP-D, and (c) most recent clinical evaluation. For the AVP-D Recovered group, additional information was collected at the time of AVP-D recovery, including the time since AVP-D diagnosis, changes in comorbidities and medications, and interval treatment of underlying hypothalamic/pituitary disease. Desmopressin doses were converted to an oral equivalent dose using a conversion factor of 10 (eg, 100 µg PO equal to 10 µg intranasal).

### Statistical analysis plan

For our retrospective case-control study in adults, we compared between-group differences (AVP-D Recovered vs AVP-D Non-recovered) in demographic data and clinical characteristics at diagnosis of hypothalamic/pituitary disease, diagnosis of AVP-D, and most recent clinical evaluation (reported as mean ± SD or median with interquartile range [IQR]). We analyzed continuous variables using a T-test or Wilcoxon rank-sum test, depending on data distribution, and categorical variables using a Chi-square test. No data imputation for missing variables was performed. We adjusted comorbidities and medications that differed by group for age, given the potential impact of age on these outcomes. We compared time to recovery based on age, sex, tumor size, initial desmopressin dose, and presence of anterior pituitary hormone deficiency at diagnosis of AVP-D using Cox proportional hazard ratio and created respective Kaplan-Meier plots for data visualization. For the three identified cases of children with recovery from chronic AVP-D, we described key clinical characteristics.

## Results

### Clinical characteristics at diagnosis of hypothalamic/pituitary disease

32 potential cases of late recovery from AVP-D were identified; of these, 18 met eligibility criteria for AVP-D Recovered. 41 potential cases of AVP-D with persistent disease were identified; of these, 36 met criteria for AVP-D Non-recovered (Fig. 1). Participants’ demographics and clinical characteristics at the diagnosis of hypothalamic/pituitary disease are summarized in Table 1. In both groups, the predominant clinical presentation leading to the diagnosis of hypothalamic/pituitary disease was mass effect, such as headache or visual field deficits. The most common hypothalamic/pituitary disease etiologies were craniopharyngioma, pituitary adenoma, and other sellar/parasellar masses. At initial diagnosis of hypothalamic/pituitary disease, participants in the AVP-D Recovered group were significantly older than those in the AVP-D Non-recovered group (*P* = .006) and more likely to have had hormone-secreting pituitary adenomas, such as corticotroph (22.2% vs 5.6%), somatotroph (5.6% vs 2.8%), and thyrotroph (5.6% vs 0%) (*P* = .05) adenomas. While AVP-D Recovered were more likely to have a documented cardiac comorbidity (*P* = .03) and be prescribed antihypertensive medication (*P* = .02), these differences were not significant after controlling for age.

**Figure 1:**
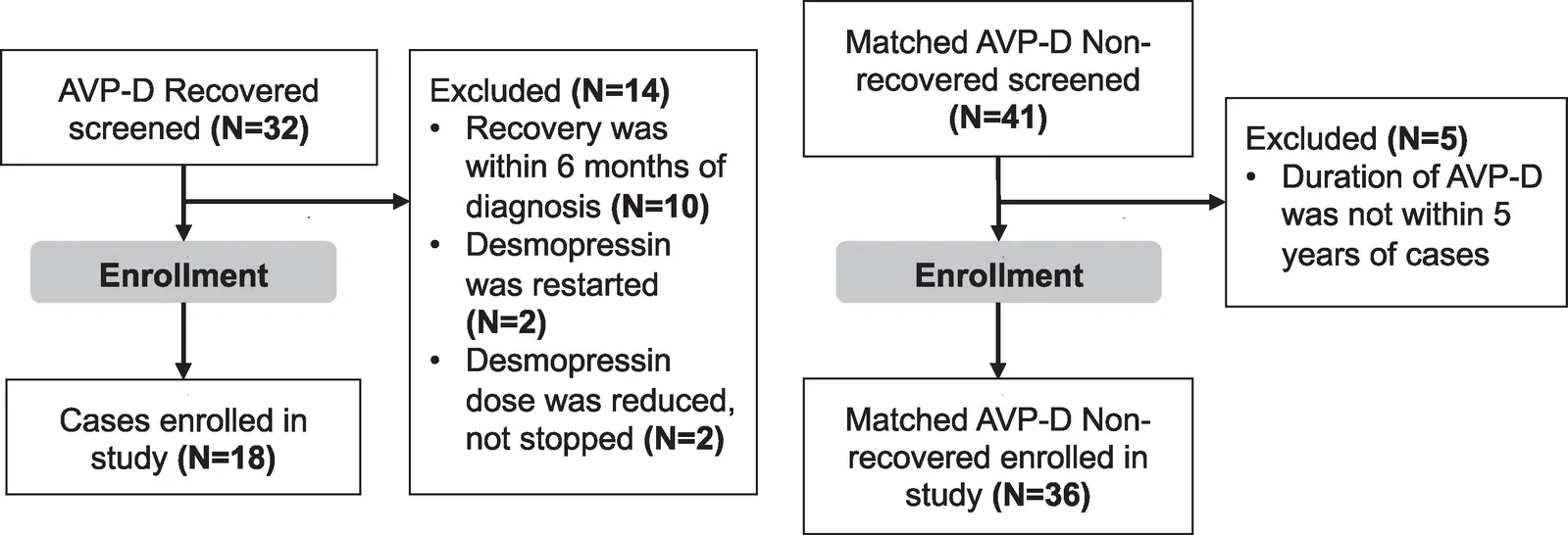
STROBE flow diagram. Strengthening the Reporting of Observational Studies in Epidemiology flow diagram of cases screened and included in this study.

**Table 1:** Demographics and clinical characteristics at diagnosis of hypothalamic/pituitary disease

	AVP-D Recovered N = 18	AVP-D Non-recovered = 36	*P*-value*
*Demographics*			
Age at diagnosis of pituitary disease (years), mean ± SD	45 ± 19	33 ± 13	.**006***^a^*
Male sex, n (%)	10 (55.6)	20 (55.6)	.9*^c^*
Ethnicity, n (%)			.50*^c^*
Black/African American	1 (5.6)	1 (2.8)	
Asian	2 (11.1)	1 (2.8)	
White	15 (83.3)	33 (91.7)	
Other	0 (0.0)	1 (2.8)	
Hispanic or Latino, n (%)	1 (5.6)	1 (2.8)	.9*^c^*
*Initial symptoms*			
Mass effect (headache, visual field deficits), n (%)	10 (55.6)	25 (69.4)	.48*^c^*
Hormone deficiency, n (%)	6 (33.3)	18 (50.0)	.38*^c^*
AVP-D, n (%)	2 (11.1)	13 (36.1)	.11*^c^*
Hormone excess, n (%)	6 (33.3)	3 (8.3)	.**05***^c^*
*Etiology of hypothalamic/pituitary disease, n (%)*			.18*^c^*
Craniopharyngioma, n (%)	6 (33.3)	13 (36.1)	
Non-functioning pituitary adenoma, n (%)	2 (11.1)	6 (16.7)	
Cushing disease, n (%)	4 (22.2)	2 (5.6)	
Acromegaly, n (%)	1 (5.6)	1 (2.8)	
Thyrotropinoma, n (%)	1 (5.6)	0 (0)	
Germinoma, n (%)	0 (0)	8 (22.2)	
Other, n (%)	4 (22.2)	6 (16.7)	
*Initial MRI findings*			
Lesion size, cm	1.9 [0.9, 3.5]	2 [1.2, 2.7]	.96*^b^*
Suprasellar extension, n (%)	4 (22.2)	7 (19.4)	.9*^c^*

Abbreviation: MRI, magnetic resonance imaging.

Comparison between participants with arginine vasopressin deficiency (AVP-D) recovery vs AVP-D non-recovery using either

*^a^* T-test,

*^b^* Wilcoxon rank sum test, or

*^c^* Chi-square.

Information is available on all 54 participants except MRI data were available for 35 participants.

*Statistically significant results are bolded.

### Clinical characteristics at diagnosis of AVP-D

Clinical characteristics at diagnosis of AVP-D are summarized in Table 2. In both groups, AVP-D most often developed after pituitary surgery (AVP-D Recovered 83.3%, AVP-D Non-recovered 61.1%) and anterior pituitary hormone deficiencies were common (AVP-D Recovered 77.8%, AVP-D Non-recovered 77.8%). At diagnosis of AVP-D, serum sodium levels tended to be lower (*P* = .06), and initial desmopressin doses were lower (*P* = .01) in the AVP-D Recovered compared to the AVP-D Non-recovered group.

**Table 2:** Demographics and clinical characteristics at diagnosis of arginine vasopressin deficiency (AVP-D)

	AVP-D Recovered N = 18	AVP-D Non-recovered N = 36	*P*-value*
*Etiology of AVP-D, n (%)*			
Post-surgical, n (%)	15 (83.3)	22 (61.1)	.18*^c^*
Post-radiation, n (%)	1 (5.6)	1 (2.8)	.9*^c^*
Pre-treatment, n (%)	2 (11.1)	13 (36.1)	.11*^c^*
*Pituitary surgery, n (%)*	17 (94.4)	31 (86.1)	.65*^c^*
Number of pituitary surgeries (mean ± SD)	1.8 ± 1.0	1.4 ± 0.8	.07*^a^*
*Hormone deficiency at AVP-D diagnosis*			
Anterior pituitary hormone deficiency, n (%)	14 (77.8)	28 (77.8)	.99*^c^*
Central hypothyroidism, n (%)	11 (61.1)	21 (58.3)	.99*^c^*
Central adrenal deficiency, n (%)	9 (50.0)	21 (58.3)	.77*^c^*
Growth hormone deficiency, n (%)	9 (50.0)	12 (33.3)	.37*^c^*
Gonadotropin deficiency, n (%)	9 (50.0)	22 (61.1)	.63*^c^*
Initial dose of desmopressin, µg/day (median [IQR])	100 [50, 100]	200 [100, 200]	.**01***^b^*
*Laboratory values*			
Untreated random plasma sodium, mmol/L (median [IQR])	146 [144, 148]	147 [146, 149]	.06*^b^*
Creatinine-based eGFR (median (IQR))	60 [60, 80]	60 [60, 88]	.76*^b^*

Abbreviations: AVP-D, arginine vasopressin deficiency; eGFR, estimated glomerular filtration rate.

Comparison between arginine vasopressin deficiency (AVP-D) Recovered vs AVP-D Non-recovered using either

^*^a^*^T-test,

^*^b^*^Wilcoxon rank sum test, or

^*^c^*^Chi-square.

Information is available on all 54 participants except: initial dose of desmopressin was available for 46 participants, plasma sodium was available for 36 participants, and eGFR for 41 participants.

*Statistically significant results are bolded.

### Clinical characteristics at the time of arginine vasopressin deficiency (AVP-D) recovery

Clinical characteristics at the time of recovery from AVP-D are summarized in Table 3. In the AVP-D Recovered group, median time from diagnosis to recovery from AVP-D was 6.3 years (IQR 1.7, 12.0, min 1, max 30). All participants ultimately discontinued desmopressin without developing symptoms of polyuria/polydipsia. Three (16.7%) participants presented with hyponatremia, with plasma sodium levels ranging from 131 mmol/L to 134 mmol/L in the context of hospitalization for one of three conditions: cardiac surgery, worsening depression, and pneumonia. Hyponatremia resolved following discontinuation of desmopressin without signs or symptoms of polyuria/polydipsia.

**Table 3:** Clinical characteristics at recovery from arginine vasopressin deficiency (AVP-D)

	AVP-D Recovered N = 18
*Duration of AVP-D, years (median [IQR])*	6.3 [1.7, 12.0]
*First signs of AVP-D recovery*	
Lack of nocturia, n (%)	12 (66.7)
Reduced desmopressin dose, n (%)	9 (50.0)
Reduced daytime urinary frequency, n (%)	9 (50.0)
Hyponatremia, n (%)	3 (16.7)
*Hormone replacement*	
Thyroid hormone replacement, n (%)	12 (66.7)
Daily dose of levothyroxine replacement, µg/day (median [IQR])	112 [100, 131]
Glucocorticoid replacement, n (%)	9 (50.0)
Daily dose of glucocorticoid replacement (hydrocortisone equivalent), mg/day (median [IQR])	15 [13, 20]
Growth hormone replacement, n (%)	3 (16.7)
Daily dose of growth hormone replacement, mg/day (median [IQR])	0.35 [0.23, 0.40]
Testosterone replacement (men), n (%)	6 (60)
Estradiol ± progesterone replacement (women), n (%)	0 (0)
*Laboratory values*	
Random plasma sodium, mmol/L (median [IQR])	141 [139, 141]
eGFR, mL/min/1.73 m^2^ (median [IQR])	72 [62, 92]

Abbreviations: AVP-D, arginine vasopressin deficiency; eGFR, estimated glomerular filtration rate.

Time to AVP-D recovery was not statistically different based on age (hazard ratio (HR) 0.99, 95% confidence interval (CI) 0.9, 1.0, *P* = .38), sex (HR 1.1, 95% CI 0.4, 2.9, *P* = .86), tumor size (HR 1.0, 95% CI 0.98, 1.04, *P* = .54), or desmopressin dose (HR 1.001, 95% CI 0.99, 1.0, *P* = .51). AVP-D recovery occurred significantly later in participants diagnosed with central hypothyroidism (HR 0.3, 95% CI 0.1, 0.9, *P* = .049) or adrenal insufficiency (HR 0.2, 95% CI 0.06, 0.7, *P* = .008) at the time of AVP-D diagnosis (see Fig. 2). Of note, all AVP-D Recovered who had adrenal insufficiency at the time of AVP-D diagnosis also had co-existing hypothyroidism. The timing of AVP-D recovery did not differ based on the presence of concurrent growth hormone deficiency (HR 0.7, 95% CI 0.3, 1.9, *P* = .5) or hypogonadism (HR 0.5, 95% CI 0.2, 1.4, *P* = .2) at the time of AVP-D diagnosis.

**Figure 2:**
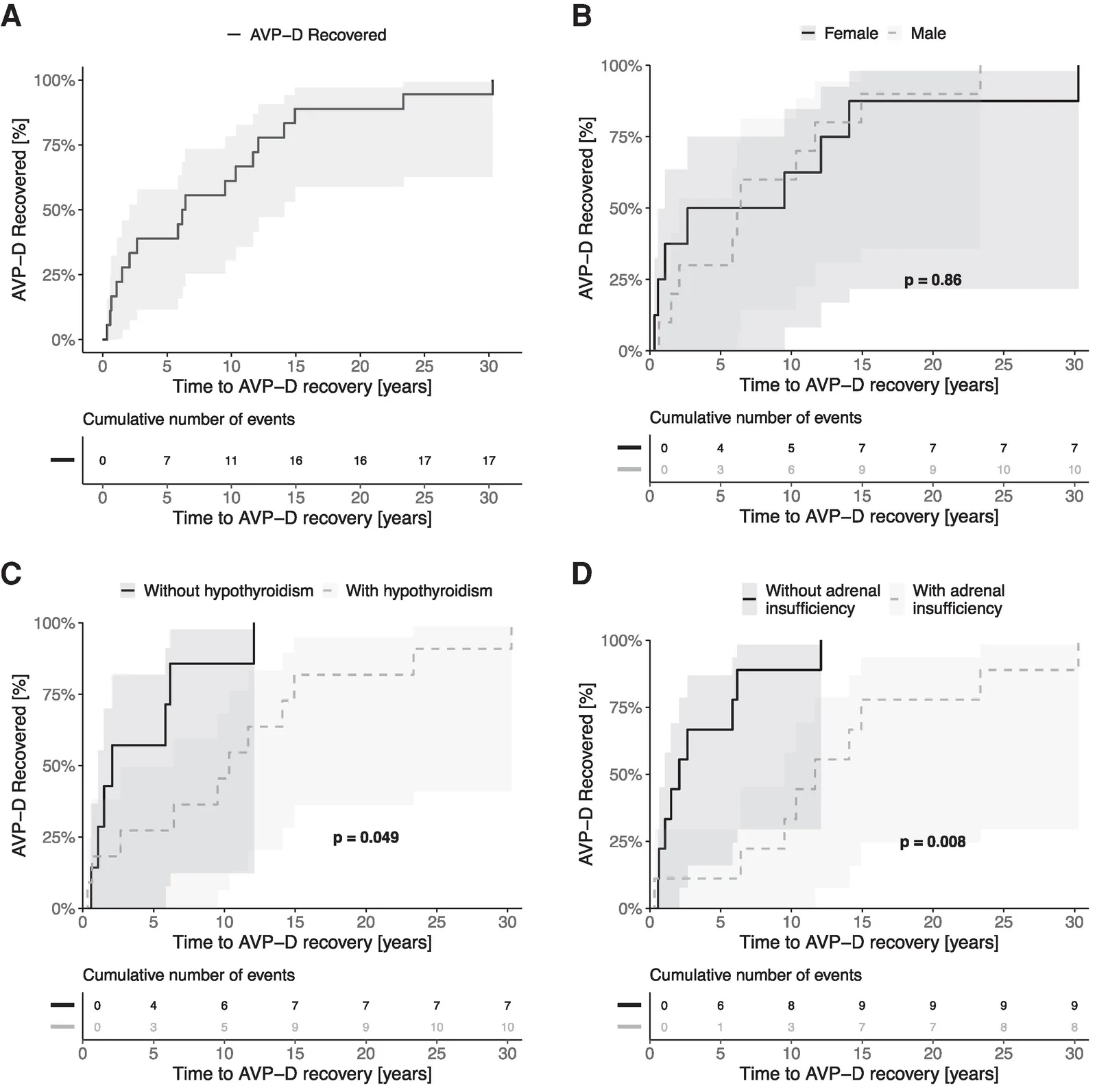
Time to recovery from AVP-D. Kaplan-Meier plots demonstrating time to recovery (A) in all participants; (B) by sex; (C) based on the presence of central hypothyroidism at time of AVP-D diagnosis; and (D) based on the presence of central adrenal insufficiency at time of AVP-D diagnosis. Median time to recovery was: overall 6.3 years, without sex differences (*P* = .86). Median time to recovery was earlier in those without central hypothyroidism (2.1 vs 10.3 years, *P* = .049) and in those without adrenal insufficiency (2.1 vs 11.7 years, *P* = .008).

### Clinical characteristics at the most recent clinical evaluation

Clinical characteristics at the most recent clinical evaluation are summarized in Table 4. AVP-D Recovered remained without clinical signs or symptoms of AVP-D relapse for a median of 5 years (IQR 3, 10, min 1, max 24). No AVP-D Recovered had experienced a recurrence of AVP-D at the time of the most recent clinical evaluation. Compared with AVP-D Non-recovered, AVP-D Recovered were less likely to have central hypothyroidism (*P* = .02) and gonadotropin deficiency (*P* = .006) at the most recent clinical evaluation. Despite these differences at follow-up, we did not observe a clear pattern suggesting recovery of anterior pituitary hormone deficiencies among AVP-D Recovered. There were no between-group differences in neuroimaging findings or comorbidities.

**Table 4:** Clinical characteristics at most recent evaluation

	AVP-D Recovered N = 18	AVP-D Non-recovered N = 36	*P*-value*
*Pituitary hormone deficiency*			
Anterior pituitary hormone deficiency, n (%)	15 (83.3)	36 (100.0)	.06*^b^*
Central hypothyroidism, n (%)	13 (72.2)	35 (97.2)	**.02** *^b^*
Adrenal deficiency, n (%)	11 (61.1)	31 (86.1)	.08*^b^*
Growth hormone deficiency, n (%)	11 (61.1)	27 (75.0)	.46*^b^*
Gonadotropin deficiency, n (%)	10 (55.6)	33 (91.7)	**.006** *^b^*
AVP deficiency, n (%)	0 (0.0)	36 (100.0)	**<.001** *^b^*
*Most recent MRI*			
No residual tumor, n (%)	6 (33.3)	18 (50.0)	.38*^b^*
Stable residual tumor, n (%)	5 (27.8)	16 (44.4)	.37*^b^*
Recurrence, n (%)	2 (11.1)	1 (2.8)	.53*^b^*
Hypoplastic pituitary, n (%)	0 (0.0)	1 (2.8)	.99*^b^*
Pituitary stalk enlargement, n (%)	1 (5.6)	0 (0.0)	.72*^b^*
Random plasma sodium, mmol/L (median [IQR])	141 [139, 142]	140 [139, 142]	.93*^a^*
eGFR, mL/min/1.73 m^2^ median [IQR]	83 [74, 98]	81 [73, 94]	.97*^a^*

Abbreviations: AVP-D, arginine vasopressin deficiency; eGFR, estimated glomerular filtration rate; MRI, magnetic resonance imaging.

Comparison between arginine vasopressin deficiency (AVP-D) Recovered vs AVP-D Non-recovered group using either

*^a^* Wilcoxon rank sum test, or

*^b^* Chi-square.

Variables were available for all 54 participants except for random plasma sodium, which was available in 53 participants, and eGFR, which was available for 50 participants.

*Statistically significant results are bolded.

### Children with AVP-D recovery

We identified three children, all boys, who recovered from chronic AVP-D. The first case was an 8-year-old boy who was diagnosed with adamantinomatous craniopharyngioma after presenting with headaches. The child underwent transsphenoidal resection, complicated by central hypothyroidism, central adrenal insufficiency, and growth hormone deficiency. Postoperatively, the child developed polyuria, polydipsia, hypernatremia (145 mmol/L), and hyperosmolality (306 mOsm/kg) with low urine osmolality (218 mOsm/kg), consistent with AVP-D, and was started on desmopressin 200 µg by mouth daily. At age 16 years, the child began to intermittently omit desmopressin doses without recurrence of polyuria/polydipsia and ultimately discontinued desmopressin, maintaining normal thirst, fluid intake, and urination. The participant has remained off of desmopressin for >5 years with normal plasma sodium and osmolality and no recurrence of polyuria/polydipsia. Pituitary hormone replacement for other deficiencies continues.

The second case was a 2.5-year-old child who presented with ataxia and was diagnosed with a low-grade glioma (pilocytic astrocytoma) involving the sellar and suprasellar region. The child underwent transsphenoidal resection, complicated by central adrenal insufficiency. Approximately 2.5 years after initial diagnosis, he developed polyuria and polydipsia during chemotherapy, with laboratory findings consistent with AVP-D, and desmopressin 100 µg by mouth daily was initiated. Two years later, upon starting treatment with targeted agents, the child developed hyponatremia while on desmopressin, prompting discontinuation of desmopressin. Since the cessation of desmopressin, the child has been eunatremic without polyuria or polydipsia for >5 years.

The third case was a child diagnosed with AVP-D secondary to traumatic brain injury at 1 month of age. During admission to the pediatric intensive care unit, the child exhibited polyuria, hypernatremia (161 mmol/L), hyperosmolality (316 mOsm/kg), and low urine osmolality (118 mOsm/kg), consistent with AVP-D. The child was managed with hydrochlorothiazide (initial dose 2 mg/kg/day divided into two doses) rather than desmopressin. Thiazide diuretics can be used to treat neonatal AVP-D in conjunction with a low renal solute diet (breast milk or certain formulas) and additional free water. The mechanism of action is not fully understood, but it is thought that thiazide diuretics can aid in proximal tubular sodium and water reabsorption (12). Treatment was discontinued after 2.5 years, and the child has remained off therapy and clinically stable for >2 years with normal plasma sodium and no polyuria/polydipsia. No other pituitary hormone deficiencies have been identified.

## Discussion

In our cohort of adults with chronic AVP-D, we found that recovery from AVP-D occurred up to three decades after onset and did not recur during decades-long follow-up, challenging the notion that AVP-D persisting beyond the initial six months is invariably permanent. Participants with recovery from AVP-D showed a trend toward milder hypernatremia prior to initiating desmopressin therapy and required lower doses of desmopressin. Recovery occurred significantly earlier in participants without concomitant central hypothyroidism or adrenal insufficiency at diagnosis of AVP-D. Those with recovery from AVP-D were less likely to have central hypothyroidism and gonadotropin deficiency at the most recent clinical evaluation, without a clear pattern of recovery of anterior pituitary deficiencies. Overall, these data are consistent with our hypothesis and suggest that less extensive damage to the hypothalamic-pituitary axis might be important for recovery from chronic AVP-D.

Hypotheses regarding the resolution of long-term AVP-D, largely derived from animal models of pituitary stalk transection, include regeneration of vasopressinergic axons, revascularization of the posterior pituitary, and formation of an “ectopic” infundibulum (6, 7, 13). Human postmortem studies have similarly demonstrated regeneration of magnocellular fibers and accumulation of neurosecretory granules within the pituitary stalk after injury (5). Notably, prior investigations have focused primarily on early recovery within six months of injury, and no studies have systematically evaluated clinical characteristics compared to a group without AVP-D recovery.

One possible mechanism of recovery from AVP-D could be the upregulation or increased sensitivity of renal AVPR2 receptors in response to chronic low AVP concentrations, eventually resulting in sufficiently concentrated urine and reduced urinary output (6). Auxiliary hormonal pathways could also play a role in the restoration of water homeostasis in AVP-D. For example, suppressed levels of apelin, a potent aquaretic peptide that counteracts AVP's action at the collecting duct, could facilitate a more efficient antidiuretic state even in the presence of subnormal AVP levels (14). Oxytocin, which shares significant structural homology with AVP, can act as a surrogate ligand for renal AVPR2 receptors. Under conditions of profound AVP-D, elevated or preserved oxytocin levels could provide sufficient cross-reactivity to stimulate aquaporin-2 translocation and mitigate polyuria (15). However, recent studies showing that AVP-D is associated with oxytocin deficiency make this hypothesis less likely (16, 17).

Initial clinical indicators of recovery from AVP-D in our adult cohort included decreased nocturia or frequency of daytime urination, or episodes of hyponatremia following continued desmopressin treatment, which prompted discontinuation of therapy. Hyponatremia thus served as an early sign of restored endogenous vasopressin function. Recovery occurred earlier among participants without hypothyroidism or adrenal insufficiency. This further supports the concept that recovery from chronic AVP-D is most likely when hypothalamic/pituitary damage is less severe (6, 18). Recovery occurred up to 30 years after diagnosis of AVP-D, which is substantially later than the few previously reported cases (eg, up to 34 months) (6). Furthermore, recovery was durable: participants remained off desmopressin for up to 24 years at the most recent clinical evaluation.

While there is greater neuroplasticity during early development than in adulthood, to our knowledge, only one prior case of recovery from AVP-D in children has been reported (9). We described three children aged 1 month to 8 years who recovered from chronic AVP-D, highlighting that AVP-D recovery may occur across the lifespan and as early as 2.5 years of age. Notably, all of our pediatric cases were boys, and future research should investigate whether girls may also experience recovery from AVP-D.

Our findings have important clinical implications. First, recovery from chronic AVP-D is possible even after long-standing disease beginning in childhood or adulthood, and the condition should therefore not be considered irreversible. Second, periodic reassessment of desmopressin should be part of lifelong follow-up to avoid overtreatment and prevent hyponatremia. Intermittently reducing and regularly withholding desmopressin doses until breakthrough may confirm ongoing dependence or unmask spontaneous recovery. Finally, given the potential for late resolution, the term chronic AVP-D more accurately reflects the clinical trajectory than permanent AVP-D, emphasizing the need for continued vigilance over time.

Strengths of this study include its case-control design with matching for AVP-D duration and international and multicenter collaboration, which enabled identification of key clinical differences between participants with and without recovery from chronic AVP-D. Limitations include the retrospective design, which precludes causal inference, and the predominantly White cohort, which may limit generalizability. Although diagnosis of AVP-D can be challenging, diagnoses in this cohort were established by expert neuroendocrinologists at pituitary centers of excellence. Furthermore, AVP-D Recovered participants were treated for a median of 6.3 years before successful withdrawal of desmopressin and remained free of clinical signs or symptoms of AVP-D during long-term follow-up. Physician practice patterns may have influenced both the recognition of AVP-D as well as the documentation of recovery, introducing selection bias. Participants in the AVP-D Non-recovered group did not undergo a standardized protocol of desmopressin withdrawal, and recovery may therefore have gone unrecognized in some participants. Formal testing was not routinely performed after cessation of desmopressin, and it is possible that AVP release remained subnormal in some participants; nonetheless, they achieved clinical recovery from AVP-D. The possibility of future recovery of participants in our control group with ongoing, chronic AVP-D cannot be excluded. Furthermore, the study design does not permit reliable estimation of the prevalence of late AVP-D recovery. Finally, although the sample size was necessarily limited by the rarity of the condition, this cohort represents the largest study to date describing late clinical recovery from chronic AVP-D.

In summary, recovery from chronic AVP-D can occur even decades after diagnosis. Compared with participants who did not recover, those who recovered were more likely to have functioning pituitary tumors and less likely to have concurrent hypothyroidism or adrenal insufficiency. These data underscore the potential for late recovery from vasopressin function through regenerative or adaptive mechanisms and highlight the importance of periodic re-evaluation of desmopressin therapy in participants with chronic AVP-D. Patients should be instructed to regularly withhold desmopressin to “break through”, and periodically trial lower desmopressin doses to assess ongoing AVP-D.

## Data Availability

Data are available upon request through the corresponding author.
